# Correlating Local Volumetric Tissue Strains with Global Lung Mechanics Measurements

**DOI:** 10.3390/ma14020439

**Published:** 2021-01-18

**Authors:** Hari Arora, Ria L. Mitchell, Richard Johnston, Marinos Manolesos, David Howells, Joseph M. Sherwood, Andrew J. Bodey, Kaz Wanelik

**Affiliations:** 1 Faculty of Science and Engineering, Swansea University, Swansea SA1 8EN, UK; r.johnston@swansea.ac.uk (R.J.); marinos.manolesos@swansea.ac.uk (M.M.); 825770@swansea.ac.uk (D.H.); 2 Faculty of Engineering, The University of Sheffield, Sheffield S10 2TN, UK; r.mitchell@sheffield.ac.uk; 3 Department of Bioengineering, Imperial College London, London SW7 2AZ, UK; j.sherwood@imperial.ac.uk; 4 Diamond Light Source Ltd., Didcot OX11 0DE, Oxfordshire, UK; andrew.bodey@diamond.ac.uk (A.J.B.); kaz.wanelik@diamond.ac.uk (K.W.)

**Keywords:** lung mechanics, micro-CT, synchrotron, digital volume correlation, alveoli

## Abstract

The mechanics of breathing is a fascinating and vital process. The lung has complexities and subtle heterogeneities in structure across length scales that influence mechanics and function. This study establishes an experimental pipeline for capturing alveolar deformations during a respiratory cycle using synchrotron radiation micro-computed tomography (SR-micro-CT). Rodent lungs were mechanically ventilated and imaged at various time points during the respiratory cycle. Pressure-Volume (P-V) characteristics were recorded to capture any changes in overall lung mechanical behaviour during the experiment. A sequence of tomograms was collected from the lungs within the intact thoracic cavity. Digital volume correlation (DVC) was used to compute the three-dimensional strain field at the alveolar level from the time sequence of reconstructed tomograms. Regional differences in ventilation were highlighted during the respiratory cycle, relating the local strains within the lung tissue to the global ventilation measurements. Strains locally reached approximately 150% compared to the averaged regional deformations of approximately 80–100%. Redistribution of air within the lungs was observed during cycling. Regions which were relatively poorly ventilated (low deformations compared to its neighbouring region) were deforming more uniformly at later stages of the experiment (consistent with its neighbouring region). Such heterogenous phenomena are common in everyday breathing. In pathological lungs, some of these non-uniformities in deformation behaviour can become exaggerated, leading to poor function or further damage. The technique presented can help characterize the multiscale biomechanical nature of a given pathology to improve patient management strategies, considering both the local and global lung mechanics.

## 1. Introduction

Interest in lung health has grown significantly recently, namely due to the COVID-19 pandemic. However, lung health commands growing concerns globally due to concerns over the long-term consequences of pollution, incidence of lung disease, and the aging population. Lung mechanics research has delivered multiscale modelling methods to study both health and pathophysiology [1]. Studies have characterized the architecture and function of the upper airways [2,3,4], the tree-like airway structure and its mechanics [5,6,7,8,9,10] with its tight coupling to the circulatory system [11], and the smallest air-containing units of the lung: the alveoli [12,13,14,15,16].

The tightly coupled respiratory and cardiovascular systems form a delicate yet highly efficient mass transfer unit. Pathogens, particulates, and other threats to the airways can alter the fine balance of mechanisms in play within this system. This can lead to an altered mechanical behaviour due to inflammation, swelling of the tissue (fluid imbalance), and altered material behaviour or tissue remodelling, to name a few. It is not feasible to clinically observe the mechanical cascade across length scales, and therefore, global measures are used to understand the overall function at the organ level. Macro-scale measures on clinical CT have been modelled to characterize disease progression [17,18], whilst other research efforts have focused on micro-computed tomography (micro-CT) [10,13,14,15,19,20]. Imaging in practice typically provides a snapshot view of the lung structure, though 4D methods are increasingly used. Global mechanical measures such as lung compliance are still more routinely used to indicate the likelihood of a given pathology.

At the alveolar level, health and disease mechanics have been studied using lung biopsies (ex vivo), isolated whole lungs (ex vivo), and whole animal models (in vivo). A wide range of imaging modalities have been used to characterize the lung geometry. Over the past 20 or so years, the use of synchrotron sources for micro-CT imaging has enabled the lung microstructure to be visualized in significant detail [12,13,14,15,19,21,22]. The high-throughput (seconds-to-minutes) and high resolution (microns) over a relatively large field of view (several millimetres e.g., 5–20 mm typically) mean detailed images can be acquired in non-fixed samples. The timescales of imaging at synchrotron facilities mean highly deformable test samples like the lung can be imaged without significant motion blur.

Studies using interrupted mechanical ventilation have shown how the airways open and expand during a respiratory cycle [13,14], enabling improved fidelity simulations of, for example, alveolar dynamics [23]. Recent works have pushed the speed of imaging to mean that long dwell (relaxation) times between loading cycles and imaging are not required [14]. This advance in methodology means that transient imaging in conjunction with digital volume correlation (DVC) measurements would enable more physiologically relevant volumetric strain fields to be computed. Such measurements characterize the local stretch of the alveoli in the context of a given respiratory cycle.

The use of image correlation methods like DVC is widespread in the field of biomechanics in hard and soft tissues [14,24,25,26,27,28,29,30,31,32,33,34]. The accuracy and physiological relevance of X-ray irradiated biomaterials (ex vivo) is consistently challenged and assessed, e.g., [24]. However, there is continual progress, both in hardware and software development, that drives the increased use of synchrotrons and DVC in biomaterial research. Here, the physiological relevance of the lung model and the experimental methodology has been further refined since Reference [14]. Although many works have used the DVC method to study various biomaterials, large straining materials like the lung have not been as widely studied using DVC. This is due to the typical time resolution required for image acquisition. This work establishes the use of DVC in lung tissue during mechanical ventilation within an intact thorax.

Previous work characterized injury mechanisms in an excised lung model, establishing lung tomography imaging feasibility at Diamond Light Source Ltd. [14,15]. The work demonstrated risks associated with ventilator-induced lung injury, particularly in the context of an already injured lung. This article presents an improved methodology for image acquisition within an intact thorax. This new model aims to deliver a more physiologically relevant strain measurement given the lungs are intact within the rib cage, i.e., with bounded expansions during a respiratory cycle. Further refinement of the fast, phase contrast tomography method implemented at Diamond Light Source Ltd. is presented here.

## 2. Materials and Methods

### 2.1. Materials

Freshly culled male CD-1 mice (age: 4–6 weeks; approximately 22–24 g) and Sprague Dawley rats (5–6 weeks; approximately 200 g) were sourced from Charles River/KWS Biotest (Bristol, UK). No live animals or human subjects were tested in this study. Samples were prepared at KWS Biotest prior to being couriered to Diamond Light Source Ltd. (Didcot, Oxfordshire, UK) within 6 h for the experiment.

### 2.2. Lung Sample Preparation

The lung vasculature was washed out with saline. The lungs were inflated with up to 0.5–5 mL depending on the size of the animal [35,36]. This was done to maintain at least their functional residual capacity and up to 50% of their total lung volume to mitigate excessive airway closure during transportation. Inflation occurred via a metal cannula inserted into the trachea. The lung was then closed off to the atmosphere using an air-tight luer stopcock to minimize lung collapse and airway degradation during transport. The cannula was held in place with sutures and tissue glue prior to being packed for shipping.

The test samples were delivered to Diamond Light Source Ltd. on the day of the testing and were dispatched and tested within 6 h of being culled. A small number of samples were presented (n = 4) due to supply chain limitations at the time. The pilot study aimed to assess the in situ mechanical test device (Controlled inflation Tester, CiT) developed for remote control at Diamond Light Source Ltd., Diamond-Manchester Branchline I13-2, and the feasibility of in situ measurements within the thorax building on previous work on excised lungs [14]. Sample numbers available during the time of the experiment were too low to perform an analysis with strong statistical significance. Therefore, this paper focuses on critiquing the rigor and capabilities of the method framework.

The cadaveric samples were carefully inserted upright into a 2-mm thin-walled polymer (PVC) cylindrical tube (70 mm diameter × 200 mm length) and the upper limbs were taped to ensure minimal movements during scanning. The head was supported to maintain an open trachea using a thread hooked under the teeth and suspended from the top of the containers. Further support was provided by tissue paper inserted around the body (outside the view of the beam) in the containers to ensure stability of the sample during rotation.

### 2.3. Lung Mechanics Measurements

Mechanical ventilation was performed on each animal to assess the mechanical state of the lung prior to imaging and to ensure that the system was airtight. To facilitate lung compliance/stiffness measurements as well as to enable streamlined measurements during synchrotron radiation micro-CT (SR-microCT), a Controlled inflation Tester (CiT) was developed.

#### 2.3.1. Controlled inflation Tester (CiT)

Knowledge of the lung’s mechanical state is critical when studying its architecture. A previous iteration of CiT used a syringe pump to deliver airflow, with pressure monitoring used to regulate lung pressure within physiological values during the imaging process [14]. To obtain continuous feedback of the lung behaviour and to ensure that the global mechanical state of the lung was monitored during imaging, a streamlined setup was established for control on the fly at the beamline.

The main component of the system is a 50-mL glass gastight syringe (Hamilton), directly driven by an encapsulated linear actuator (Nanotec L4118L1804-T6X2-A50) and held within a custom 3D-printed housing structure. The actuator (resolution 0.01 mm/step) can be used to control the delivered volume, with correction applied for compressibility of the air. This calculation depends on the pressure, measured with a silicon micromachined amplified pressure sensor (Omega PXM319, Omega, Manchester, UK), and the initial system volume. A photodiode (Osram Opto BPW 21, OSRAM Opto Semiconductors GmbH, Regensburg, Germany) and LED (Cree C503 Series, RS Components Ltd., Corby, UK) were placed on opposing sides of the syringe to provide a reference position for the syringe to automatically return to the initial volume. A 3/2 solenoid valve (SMC VDV valve) was used to allow automatic zeroing of the pressure to atmosphere.

The system can be operated in manual volume control mode, or can run automated Pressure-Volume (P-V) loops by setting the upper and lower limits on P. All hardware was controlled and measured using a National Instruments Data Acquisition card (USB-6211) and the control and acquisition software was written in LabVIEW (National Instruments, Austin, TX, USA).

**Figure 1:**
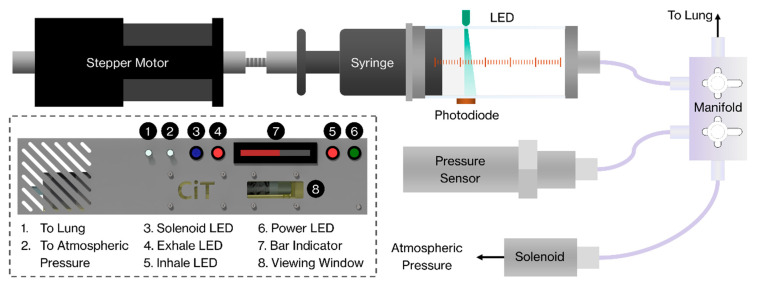
Schematic of the Controlled inflation Tester (CiT) hardware and its functionality.

**Figure 2:**
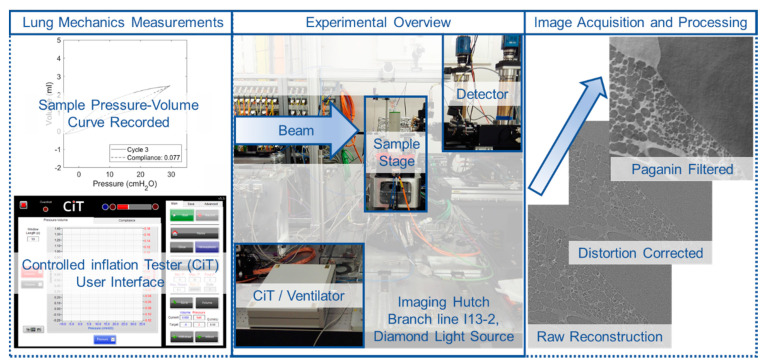
Experimental overview including the in situ ventilation setup on the beamline at branch line I13 Diamond Light Source: lung mechanical measurements acquired using the custom-built Controlled inflation Tester (CiT) for each scan are illustrated alongside the postprocessing steps performed for each image.

The hardware uses a stepper-motor, syringe, and pressure transducer to mechanically load the lung in a similar fashion to a syringe pump (Figure 1). Speeds to infusion/withdrawal ranged from 50 to 200 mL/min during testing and setup. Additional functions can be performed including the use of solenoid valves to switch to atmospheric pressure (mitigating unwanted formation of vacuums in the system during test setup or zeroing); automatic compliance corrections for the connections between the lung and the device; variable ventilation controls, e.g., speed, volume, and pressure of inflation; and start and stop criteria either predefined or based on cycle characteristics computed on the fly. The control functionality is programmed in LabView, with a streamlined interface recording test data (see Figure 2).

#### 2.3.2. Protocol for Pressure-Volume (P-V) Measurements during SR-micro-CT Imaging

The nature of mechanical ventilation was a simple steady, linear ramp infusion, and withdrawal using CiT. Mechanical ventilation was performed purely to acquire a measure of lung compliance/stiffness and to mechanically deform the lung prior to each tomography image. Lung compliance/stiffness was obtained through cyclic loading under pressure control. The samples underwent at least 10 cycles of continuous ventilation until lung compliance stabilized (residual error < 1%). During mechanical ventilation, airways which may have collapsed reopen and the tissue (after a period of inactivity) also must adapt to movement once again. There is considerable change to the lung compliance during this time, which requires reconditioning. Therefore, a control mode within CiT was developed to map the stability of a given P-V loop. If the P-V loop begins to stabilize, the slope becomes more similar from one cycle to the next cycle, and therefore, the error between cycles reduces towards negligible magnitudes. There are other sources of error that may arise when setting up such lung experiments, e.g., leakage. If there is a leak, the leak would be detected, as the P-V loop would not stabilize between cycles and the residual error would remain high.

Cycling prior to imaging ensured that specimens had the same base mechanical performance. Once ready to begin imaging, 2–3 cycles were completed and the cycling stopped at a designated pressure. After 30 s, the tomogram was acquired. This delay enabled any reorganization of air within the lung and partial relaxation of the tissue to occur. This delay was optimized to mitigate motion blur during the collection of each tomogram. The target and actual end pressures during image acquisition were recorded. The true mechanical loading condition during an interrupted test is important and must be recorded in full. Therefore, the CiT continued to record pressure changes within the lung during the imaging cycle to give the context of the mechanical state to the resultant tomogram. This process was repeated, with each sample being ventilated prior to each scan.

### 2.4. Image Acquisition

Tomography was performed at the Diamond-Manchester Imaging Branchline I13–2 [37,38] of the Diamond Light Source synchrotron (Oxfordshire, UK). A partially-coherent, near-parallel, polychromatic “pink” beam (circa 8–30 keV) was generated by an undulator in an electron storage ring of 3.0 GeV voltage and 300 mA current. For data collection, the undulator gap was set to 5.0 mm. The beam was reflected from the platinum stripe of a grazing-incidence focusing mirror and high-pass filtered with 1.3-mm pyrolytic graphite and 3.2-mm aluminium, resulting in a beam of weighted-mean photon energy at approximately 27 keV. Samples were aligned for data collection under low-dose conditions (approximately 2 min per sample) by temporarily setting the undulator gap to 7 mm. Slits were used to crop the beam just outside the field of view; this limited both the sample exposure and the intensity of noise arising from scintillator defects. Test samples, within their containers, were connected with a remotely controlled airline to CiT and mounted on perpendicular Newport MFA-PPD (Newport Corp., Irvine, CA, USA) linear stages atop an Aerotech ABRT-260 (Aerotech Inc., Pittsburg, PA, USA) rotation stage. Various propagation distances were trialled, and approximately 70 mm was chosen to give an adequate level of phase contrast; 1201 projections were acquired at equally spaced angles over 180° of continuous rotation (“fly scan”), with an extra projection (not used for reconstructions) collected at 180° to check for possible sample deformation, bulk movements, and beam damage relative to the first (0°) projection. Projections were collected by a pco.edge 5.5 Camera Link (PCO AG, Kelheim, Germany) detector (sCMOS sensor of 2560 × 2160 pixels) mounted on a visible light microscope of variable magnification. Magnification was controlled via rotation of a turret incorporating various scintillator-coupled objective lenses. A 1.25× objective (used for the main study) was coupled to a CdWO_4_ scintillator, mounted ahead of a 2× lens providing 2.5× total magnification, providing a field of view of 6.7 mm × 5.6 mm and an effective pixel size of 2.6 μm. A 2× objective (used for a second measurement), coupled to a CdWO_4_ scintillator providing 4× total magnification, achieved a field of view of 4.2 mm × 3.5 mm and an effective pixel size of 1.6 μm. A previous work [14] showed that scan times under 30 s were required to ensure stability during imaging (in excised samples). There is a balance between speed and signal–noise for such highly deformable samples. Multiple projection and exposure settings were assessed. Deformations were deemed acceptable for scan times up to 100 s with the new ventilation control system (CiT) in low lung volumes. At higher lung volumes, the lung has greater recoil. Therefore, a balance of sampling (number of projections) and scan time was established. The magnifications were chosen to reliably capture the same sub-volume of a highly deforming lung through a respiratory cycle, with sufficient spatial resolution to discern the airway walls. An exposure time of 30 ms was chosen. This led to scanning times of circa 36 s per sample, sufficient to capture the main features without motion blur. Prior to each scan, each specimen was cycled between −5 and 30 cmH_2_O using CiT and then stopped at the desired pressure for that scan. This process of ventilating with CiT, stopping at a given pressure and imaging, was repeated to acquire multiple images to capture the deformed geometry at various points along its P-V curve.

### 2.5. Image Analysis: Reconstruction and Filtering

Data were reconstructed via filtered back projection with the open source, modular pipeline Savu [39,40]. Projection images were subject to flat- and dark-field correction, followed by a correction for optical distortion [41] (Figure 2) and another correction to suppress ring artefacts [42]. Phase retrieval of inline phase contrast scans was performed with Paganin filtering to restore quantitative detail to tomograms and to facilitate straightforward threshold-based segmentation of tissue from air. The degree of Paganin filtering is proportional to the relative contribution of X-ray absorption (β) and phase shift (δ) to projections. Under-filtering confers marginal benefit for segmentations, but over-filtering leads to feature loss and blurring. A range of filtering degrees was screened, and δ/β = 1000 gave the clearest results (Figure 2). These settings showed no observable loss relating to the volume/mass calculations (<0.01%) of the sample within the image when segmenting the Paganin-filtered plus distortion-corrected image versus the solely distortion-corrected image. Digital volume correlation (DVC) works by tracking displacements, deformations, and distortions of groupings of voxels forming larger features (>50 µm). Any texture differences shown within the tissue walls are averaged out in the analysis and would otherwise introduce noise; therefore, filtering was considered acceptable for all images. The influence of filtering on the DVC analysis is quantified as part of the zero-strain test.

### 2.6. Image Analysis: Digital Volume Correlation (DVC)

DVC analysis was performed using DaVis v10.05, LaVision, Göttingen, Germany. Images were downsized to 16bit *.raww images, with the background segmented and removed. A sequence of images was collected for each sample and processed in DaVis. Full-field 3D strains of each lung sample were computed with sub-volume sizes screened down to 32 voxels. Each strain stage relates to a point on the P-V loading curve. Small movements of the sample can occur during the entire test duration (approximately 30 min), causing rigid body motion of the sample from the first volume image to the last. The DaVis software can remove any rigid body motion detected prior to computing the strains. Zero-strain tests were performed, computing strains from repeat scans acquired at the same loading condition, in addition to the main study. The zero-strain test aimed to quantify the degree of uncertainty in the strain measurement. The lung sat on the stage under its own weight, with its own elasticity and within a compliant structure (sample container). Therefore, the zero-strain test provided a measure of confidence in the strain measurements produced during the loading cycles (the main experiment). Much research is performed in uncertainty analysis with DVC [43]. The previous study in the highly deformable lung samples demonstrated that <5% strains were observed throughout the volume [14].

## 3. Results

### 3.1. Imaging Optimisation

**Figure 3:**
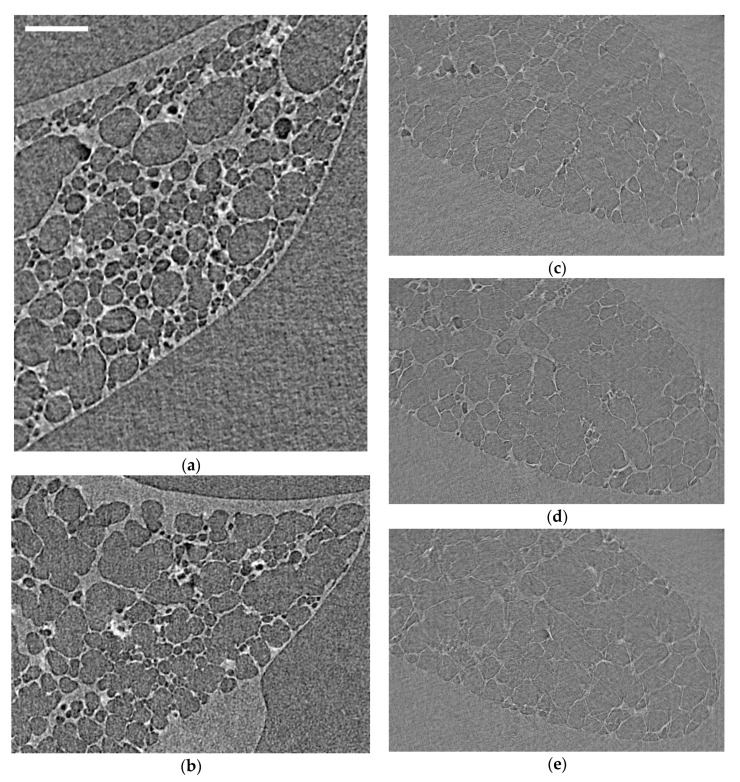
Sample raw reconstructed images from mouse and rat lungs under different states of motion to illustrate the differences between static and dynamic images when optimizing dwell times prior to scanning: (**a**,**b**) mouse lung at low inflation state (0 cmH_2_O) and essentially static; (**c**) rat lung recently inflated; (**d**) rat lung continuously inflated slowly with air during the scan (approximately 0.1 mL); and (**e**) rat lung cyclically inflated/deflated by 0.1 mL during the scan. The image scale bar (top left) indicates 200 µm for all images in the figure.

The first stage of the experiment involved optimizing imaging parameters. Sample images were taken in different sub-volumes of the rodent lungs (Figure 3), with key features and imaging artefacts highlighted. When ventilating lungs for imaging (i.e., unfixed samples), timing is important to enable air to settle and to reorganize within the structure, allowing for tissues to relax to a certain extent and stopping significant motion during imaging. Increasing the scan speed can offset any risk of motion; however, it can also lead to poor signal-noise. Whilst optimizing such timings, the images were taken at different stages of motion to see whether lung motion can be mitigated and where motion blur artefact in the images can be minimized. Figure 3c–e specifically shows the lung whilst deforming. Here, there is intentional movement during the test to exaggerate the motion blur in the image. This firstly helped calibrate visual perceptions for the finer degrees of motion blur (much like focusing a camera). However, a secondary outcome was to potentially illustrate airway deformation patterns that observed during a single fast scan (<25 s). Much work is needed to extract any intelligible and quantifiable information from these images. However, the degree of motion towards its peripheries in Figure 3e indicates the potential for better ventilation in regions of increased motion blur and/or less deformation/ventilation in central regions, which appear more “in focus”. This was a secondary observation made during imaging optimization when compared to the primary study performing DVC to characterize local tissue strains. The main point of this exercise was to determine appropriate image acquisition and sample dwell times for the interrupted test and to best capture the lung geometry at various states of inflation. The images presented in Figure 3 led to the final protocol/timings outlined in Section 2.

### 3.2. Zero-Strain Test and the Reliability of the DVC Results

**Figure 4:**
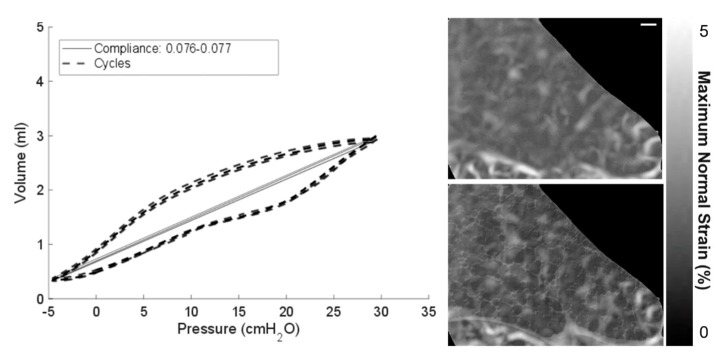
Repeatability of mechanical ventilation (4 cycles shown) and the zero-strain test Digital volume correlation (DVC) results performed at 20 cmH_2_O are shown. A P-V curve for each cycle is overlaid on the same plot (left) plus the DVC strain fields for the zero-strain test (right): the unfiltered images (top right) and Paganin-filtered images (bottom right), with both showing strains below 5%. The image scale bar (top right) indicates 200 µm for all images in the figure.

Further analysis of image postprocessing techniques and the resultant impact on DVC outputs is given in Figure 4. Once imaging parameters were set, a sample was mounted and ventilated until compliance converged on a given value (i.e., the airways had all reopened, and all air had reorganized itself within the lung). The P-V curve in Figure 4 illustrates the repeated cycling performed prior to each test. The classical sigmoidal form of the curve is observed. P-V measurements were monitored prior to each scan to assess any stiffening or damage sustained by the sample either due to degradation (given that these are cadaveric samples) or due to beam damage (radiation/heating). This is the first test for reliability for any subsequent strain measurements.

The second test is on the repeatability of imaging the same region under the same state of deformation, where strain should be negligible: the zero-strain test. The given images are taken of a sub-volume within the thorax of a highly viscoelastic and deformable structure; it is envisaged that some motion is unavoidable and that the exact beam path may differ from scan to scan. To quantify the expected error, a zero-strain test was performed at a relatively high level of inflation. A higher level of inflation was chosen since this is where the lung is very likely to want to contract and return to a less deformed state (therefore highlighting a worst-case scenario for unwanted deformations from scan to scan). The images were taken within a minute of each other, and the resultant DVC measurement is shown in Figure 4. Within the bulk tissue, there is a relatively low level of strain computed <3%. Towards the peripheries, strains approach 5%. This is where larger areas of the image are formed of only soft tissue with few discernible features (i.e., no alveoli). Tissue/air interfaces seen in the alveoli, which are readily distinguishable and tracked by the DVC algorithm, are the focus of this study. It is understandable that a higher range of strains may be computed in the relatively feature-free solid tissue or background, since these are the noisier parts of the image with fewer features to track. Edges and boundaries are kept in the reported images as a reference to the lung surface. However, it is noted that the boundaries, where large movement occurs, may also be more susceptible to error. This error (<5%) is however very small compared to the magnitude of tissue deformation during a breathing cycle: approximately 100%.

The third test of reliability of the DVC results is in the use of image postprocessing prior to performing DVC computations. Figure 2 highlights the clarity of images when distortion corrections and Paganin filters were used on the raw reconstructed data. Figure 4 shows two strain plots: the top shows the distortion-corrected images used to compute the strain field, and the bottom shows the Paganin-filtered distortion corrected images used to compute the strain field. The two sets of images with their strain plots overlaid are shown in Figure 4. The contrast of tissue and air is observably clearer in the Paganin-filtered images and the impact on the resultant strain field is minimal. The magnitudes and distribution of strain fields are comparable for both the distortion-corrected images and those with additional Paganin filtering. The results in this paper will focus on the filtered images as there are fewer sharp localizations in the strain. Such localizations are potentially caused by noise present within the tissue boundaries, mentioned earlier, which are reduced by filtering. The final consideration for reliability of the data lies in the use of the same sample to acquire and compute multiple deformation steps within a breathing cycle. Any degradative factors having to do with the biology or beam influence and how to quantify such potential factors are considered.

**Figure 5:**
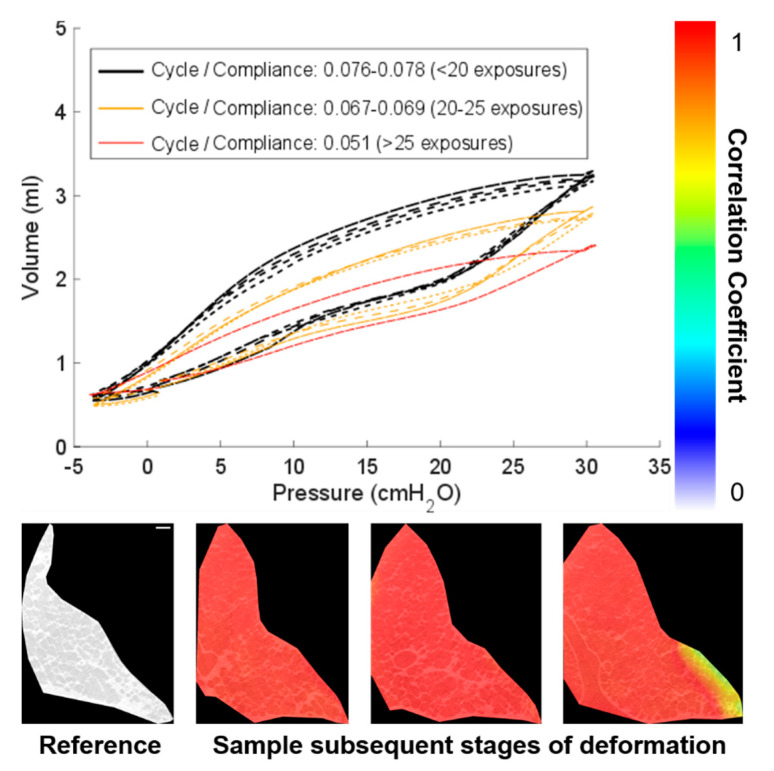
Sample degradation after 20 exposures indicated by a drop in compliance plus the lowering of correlation quality where large deformations occur: P-V curves show the stability of the lung compliance and the gradual degradation over time. The image scale bar (bottom left) indicates 200 µm for all images in the figure.

Repeat scans were taken of each sample at different stages of deformation within a given P-V cycle. Earlier, Figure 4 highlighted the potential errors that may arise in strain measurement towards the boundaries particularly where large deformations are expected. Here, these errors were minimized (given that the zero-strain test resulted in strains <3% for most of the region of interest). Smaller increments in strain (from image to image) may mitigate errors associated with large deformation experiments. This may not always be possible with highly deformable test samples such as lung tissue and its nonlinear characteristic behaviour. Figure 5 illustrates the drop in correlation coefficient where large jumps in deformation occurred from image to image.

**Figure 6:**
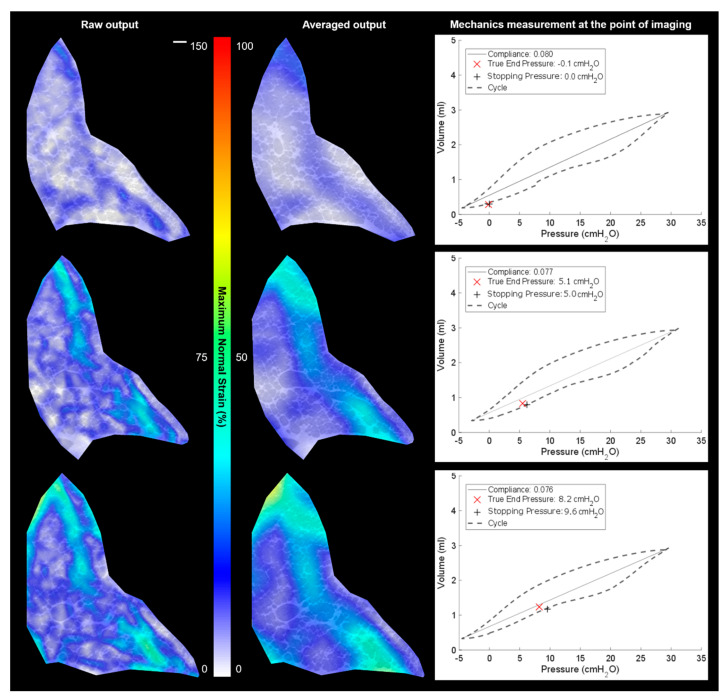
Results overview of the pressure cycles between −5 cmH_2_O and 30 cmH_2_O with stopping pressures at 0 cmH_2_O (**top row**), 5 cmH_2_O (**middle row**), and 10 cmH_2_O (**bottom row**): the DVC results are shown with detailed strains (**left**), with mild averaging of 7 local data points, plus the same strain field averaged over 15 local data points (**centre**) to illustrate the averaged behaviour in the region, plus mechanical data for each run including the true end pressure recorded during the scan and the sample compliance. The strain peak is around 60% in these colour contour maps. The image scale bar (**top left**) indicates 200 µm for all images in the figure. The images are of a single representative specimen at multiple inflation states.

**Figure 7:**
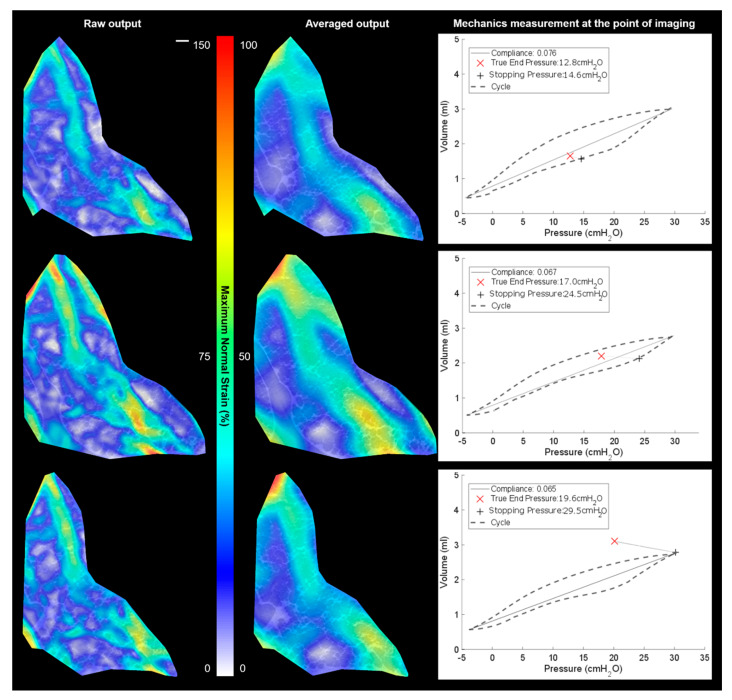
Results overview of the pressure cycles between −5 cmH_2_O and 30 cmH_2_O with stopping pressures at 15 cmH_2_O (**top row**), 25 cmH_2_O (**middle row**), and 30 cmH_2_O (**bottom row**): the DVC results are shown with detailed strains (**left**), with mild averaging of 7 local data points, plus the same strain field averaged over 15 local data points (**centre**) to illustrate the averaged behaviour in the region, plus mechanical data for each run including the true end pressure recorded during the scan and the sample compliance. A strain peak >100% is shown in these colour contour maps. The image scale bar (**top left**) indicates 200 µm for all images in the figure. The images are of a single representative specimen at multiple inflation states.

The sample is a cadaveric sample, and overtime, the lung will degrade in mechanical properties. Figure 5 highlights the repeated cycling and number of exposures that this sample observed. After 20 exposures (>6 h after the sample was collected), the compliance started to drop. The degree of control over the lung volume also degraded towards the end of the experiment, and as a result, small adjustments to the mounting of the sample were needed to minimize motion and to maintain clear airflow. The DVC correlation coefficient, highlighted in Figure 5, for the latter scans dropped from >95% for the data presented in Figure 6 to having regions significantly below this threshold for data shown in Figure 7 (again, towards the boundary for large deformations). Therefore, this paper will discuss the key results in the context of the reliable data collected early in the experiment where compliance was stable and repeatable (Figure 6) and where caution may be needed when interpreting the data as compliance started to decrease (Figure 7) due to degradation or beam damage. This reinforces the need for continuous monitoring of the mechanics during imaging used here to provide context for the resultant imaged microstructure.

### 3.3. Local vs. Global Mechanics Highlighted by DVC and P-V

Figure 6 and Figure 7 both show two types of strain plot for one slice in the 3D volumes alongside P-V curves for each scan (strain map). During imaging, the ventilation cycling was stopped and held at a given pressure; 30–60 s was the dwell time between ventilation and the scan to enable the air to redistribute and the tissue to settle into position for that target, stopping pressure. During the dwell time, the pressure did drop as expected due to potential increase in airway volume as airways stretch and move to accommodate air pressure. This true end pressure is noted as the pressure during the given scan (highlighted in each plot).

The two strain plots in Figure 6 show the detailed strain and the averaged regional strain (with deformation vectors averaged over a larger area). Before each image was taken, a new P-V loop was acquired to ensure consistent reporting of the mechanical integrity and to reach the next desired stopping pressure. The averaged strain plot helps highlight the general trend of the airways stretching just infield from the boundary pushing outwards in a tensile fashion. Each neighbouring airway is connected by shared membranes, meaning that there is significant interdependence in mechanical behaviour. Regions that are well ventilated impinge on the space of those inflating less, causing tensile strain differentials across the lung volume. The plots show that the strains peak locally around 25% up to the 60% mark when inflating from 0 cmH_2_O to 10 cmH_2_O. Note that raw output data highlights more local deformations, which are higher/lower than the averaged plots. This is indicative of the local heterogenous behaviour observed in this heterogenous airway structure.

Figure 7 sees this general trend of a largely deforming airway region inset from the boundary continue. The top image, taken whilst still maintaining consistent compliance with previous scans, peaks its strains around the 80% mark for the 15 cmH_2_O measurement. However, after this, the compliance drops slightly. The general trends of major deformation locations remained consistent for the 2nd image in Figure 7, with strains reaching around the 100% mark. However, the apparent larger deformed final image had clearly lost stability and did not maintain a good volume of air, showing that strains drop just below 100% and that the true end pressure dropped more rapidly prior to the scan. This is where the test ended for this sample.

## 4. Discussion

**Figure 8:**
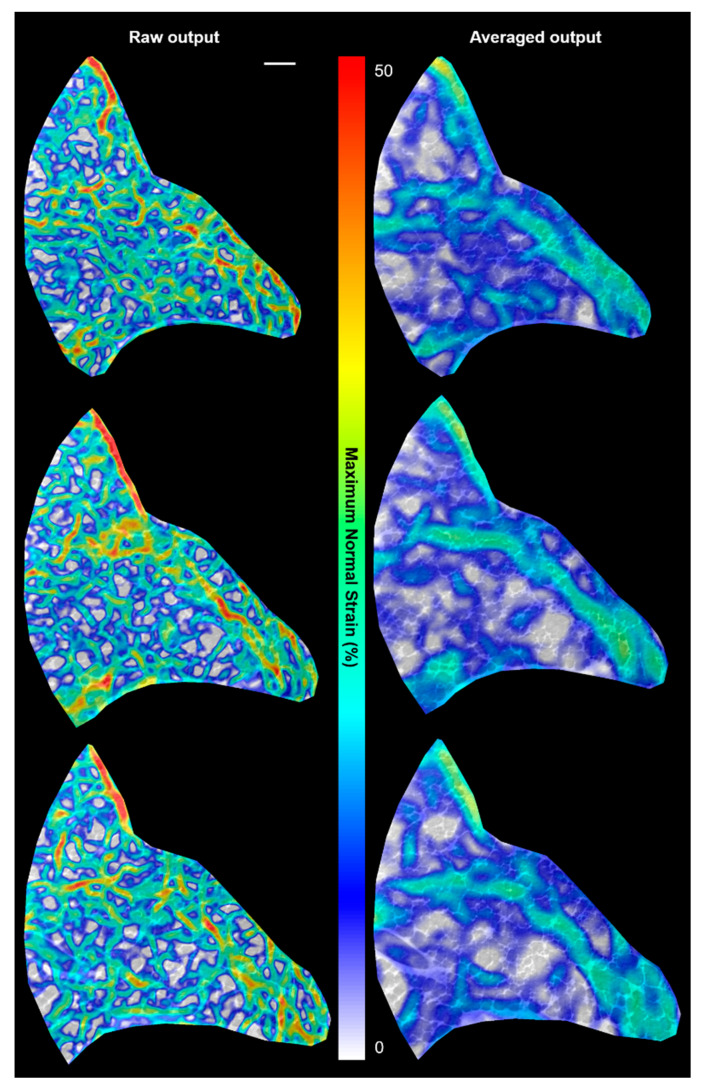
Sample results from higher magnification lung ventilations (2× compared to 1.25×): the higher magnification enables more detailed strain fields approaching the scales of the alveolar walls to be resolved. The image scale bar (**top left**) indicates 200 µm for all images in the figure.

Focusing on the established as trustworthy strain data from Figure 6 (and part of Figure 7), regional differences in how a lung deforms during respiration have been visualized. DVC was performed to characterize local stretch in lung tissue, acquired during several inflation points during a respiratory cycle. It is well-known that the local heterogeneities within the lung structure, composition, and airway resistance contribute to the complex asymmetric deformations during breathing. The DVC data shows clearly regions inset from the boundary expanding more significantly than more central regions (with respect to the airway source, i.e., trachea, towards the “left” of each strain plot). Given the contrast of air and tissue being tracked to compute the strains, more refined strain maps were not considered here. Finer DVC computations may be more susceptible to noise given smaller features than the walls (few microns wide) are not discernible in the image for tracking. Further study at higher magnifications instead is recommended, as shown in Figure 8. There is always the trade-off of visualizing a larger volume (for the global overview) vs. improved resolution (local detail). However, it was shown in Figure 5 that approximately 20 exposures did not see significant loss in lung mechanics; therefore, there is potential to run multiple magnifications sequentially to map the lung deformations across length scales within a given sample.

The higher-magnification images in Figure 8 enable strain fields approaching the wall dimensions to be produced. These too can be averaged to demonstrate the same global observation of greater stretch towards the lung boundary. Further contrast enhancement of the alveolar walls may be achieved with perfusion of a contrast agent into the microvasculature, and this would enable intra-tissue strains to be potentially evaluated. This experiment demonstrated the ability to reliably track phase contrast between the air–tissue barrier within the intact thorax. Multiple strain states (i.e., degrees of ventilation) are also computed here to enable a range of strains up to 100% to be reliably mapped compared to previous work with injured lungs showing a single instantaneous strain state in excised lungs [14]. The intact thorax provides a degree of protection from premature beam damage, enabling more strain states to be imaged in the same region (compared to an excised sample). The imaging session with continuous P-V monitoring provided indicators of where reliability in the resulting computed strains may be lost (i.e., when the specimen starts to degrade).

Further extensions of the DVC analysis can include correlating local deformations with total volume changes in the specimens and assessing how closely these relate to the global lung P-V measurements. This could help assess degrees of compressibility/expansion within the tissue and capture absolute distributions of volume across the whole lung. Given that sub-volumes of lung were imaged here, it is not straightforward to perform such a calculation. However, work is ongoing to study smaller lung volumes and larger fields of view optics, where the total lung can be imaged across all states of inflation. Then, a direct comparison on the volume measurements can be made in addition to links with global compliance and comparative distributions of ventilation volumes from region to region, presented here.

One compromise of imaging through the thorax is the reduction in signal–noise, which can lead to a loss in image clarity. The postprocessing for distortion corrections and Paganin filtering is shown to draw out the lung architecture clearly in Figure 2. The zero-strain test showed some softening of strain maxima for the Paganin-filtered images compared to the purely distortion corrected images. This again helped mitigate issues relating to imaging sub-volumes within a thick specimen (approximately 70 mm dia), where poor signal–noise may be expected particularly for very short exposures (30 ms). Furthermore, when using phase contrast imaging, there is the risk of generating shadows around features which can create sharp boundaries of dark and light within the greyscale image. It may seem preferential to create this shadow of dark around the boundary to help with resolving that boundary. However, the detail of the boundary location can be lost in this region and may also lead to apparent broken boundaries between the tissue and air. In any postprocessing, such as segmentation or DVC, such artefacts can cause errors and require considerable manual processing to mitigate such errors. There is therefore always a trade-off of resolving the feature by increasing phase contrast, whilst minimizing artefact. Paganin filtering enables the contrast to be rebalanced at each boundary to minimize errors in detecting the boundary potentially caused by shadows and light bleeding across the boundary. The filtering assisted with denoising in regions that were featureless for contributing to the DVC computation enabled tissue and air to be readily discernible.

The development of this framework to study lung deformations using DVC is extremely powerful. Although the use of such imaging techniques may not be new [12,13,14,15,17,18,19,20,21,22,23], the ability to register reliably the in situ mechanics alongside the images has streamlined its potential use in lung mechanics research. Work is continuously progressing to improve the method, to enable faster imaging to mitigate motion artefact in unfixed samples, for instance. When imaging speeds increase, continuous tomography imaging in highly deformable tissues like the lung will be possible. In Figure 3, the current limits of imaging speed with the current hardware are demonstrated, showing different degrees of motion blur during continuous imaging and in situ loading. Recent works on stiffer viscoelastic solid biomaterials demonstrated the feasibility for continuous synchronous mechanical loading and imaging [44]. With lung tissue, the deformation magnitudes are much greater than the biomaterials in Reference [44], over a shorter time period; therefore, considerable motion blur is observed (as demonstrated in Figure 3). When imaging frequency increases >1–5 kHz (compared to the 100 Hz used here), then continuous loading and imaging in such highly deformable tissues is possible. This would negate the need for interrupted loading cycles (i.e., partial relaxation) and provide a more representative view of the lung deformations present in breathing. An intermediate step to achieve this goal of continuous monitoring is to link surface mapping techniques such as digital image correlation with volume methods. Our team has extensive experience with these surface strain measurement techniques [45,46,47,48,49,50,51,52], and recently, groups have published studies exploiting the technique on lungs during mechanical ventilation [53]. Coupling and correlating imaging modalities can lead to more complete overviews of the mechanics and function without the need to compromise on the in situ loading regime. This article however demonstrated the first steps to achieving such results within an intact thorax.

The potential to study pathological lungs to derive an understanding of the local mechanics in the context of an overly stiff lung, for instance, is extremely useful. As previously discussed in [14], when considering patient management strategies, it is a challenge to mitigate further damage or collapse of airways. However, detailed strain maps shown here, coupled with global measures will enable detailed validation of computational simulations of patient management strategies. Obtaining a feeling for global pressure loading (P-V, or ventilator strategy, for instance) and its impact on the alveolar scale will enable the refinement of multiscale simulations in lung mechanics and assist in the development of protective lung management strategies. The next phase of this work will study healthy and pathological lungs in a larger-scale study to enable more quantitative parameters to be derived for modelling purposes, while the experimental technique was established here.

## 5. Conclusions

A methodology for in situ mechanical ventilation of rodent lungs with SR-micro-CT was presented. Multiple facets of experimental technique and the key considerations required, when establishing such experiments, were assessed. The study showed the local variability in strain fields observed within the lung tissue during a breathing cycle. The distinct patterns of deformations highlighted the contrast in degree of tensile strains between peripheral regions of the tissue compared to central regions. Local heterogeneous structure and its resultant mechanics were captured. The technique advanced on previous methods in terms of real-time control and monitoring during imaging, providing both improved confidence and context to the measurements.

## Data Availability

Data available via www.aroraresearch.com.
